# Warm Versus Cold Irrigation in Brain Surgery: A Comprehensive Review

**DOI:** 10.7759/cureus.109145

**Published:** 2026-05-18

**Authors:** Allison Shay, Heather K Johnston, Akshar G Patel, Louis Reier, Mohammad Arshad, Javed Siddiqi

**Affiliations:** 1 Department of Clinical Sciences, St. George's University School of Medicine, St. George's, GRD; 2 Department of Medical Education, California University of Science and Medicine, Colton, USA; 3 Neuro Intensive Care Unit, Arrowhead Regional Medical Center, Colton, USA; 4 Department of Neurosurgery, Graduate Medical Education, Arrowhead Regional Medical Center, Colton, USA; 5 Department of Neurosurgery, Desert Regional Medical Center, Palm Springs, USA; 6 Department of Neurosurgery, Riverside University Health System Medical Center, Moreno Valley, USA

**Keywords:** cold irrigation, hypothermia, intraoperative thermoregulation, irrigation temperature, neuroprotection, selective brain cooling, warm irrigation

## Abstract

Irrigation is used in every brain surgery operation, yet the optimal temperature of irrigation fluid remains poorly defined and is a topic of debate among neurosurgeons. The majority of neurosurgeons who use warm irrigation cite maintaining normothermia and its perceived hemostatic benefit as their main reasons. However, basic science, neurophysiology, and clinical data challenge the rationale for warm irrigation and suggest that cold irrigation is more advantageous given its neuroprotective properties. Hyperthermia exacerbates neuronal injury, increases metabolic demand, lowers seizure threshold, and worsens outcomes following CNS injury. Conversely, hypothermia reduces intracranial pressure, decreases cerebral metabolism, limits oxidative damage, and improves outcomes in numerous injury models. This review synthesizes the available literature on irrigation temperature in cranial neurosurgery and argues that cold irrigation is superior because it is physiologically consistent with the principles of neuroprotection, metabolic suppression, and seizure prevention.

## Introduction and background

Intraoperative irrigation serves multiple purposes in neurosurgery: clearing blood from the operative field, keeping brain tissue well-hydrated and preventing cortical desiccation, improving visualization, and facilitating atraumatic tissue handling. Despite its ubiquity, little consensus exists regarding the optimal temperature of irrigation. Most operating rooms default to using warm irrigation (typically defined as 37°C or higher), a convention influenced by general surgery practice to help prevent the development of systemic hypothermia [1].

However, the brain is uniquely sensitive to temperature. Hyperthermia worsens outcomes after ischemic, hemorrhagic, and traumatic brain injury [2-4], while mild hypothermia confers robust neuroprotection through reductions in metabolic demand, membrane stabilization, modulation of inflammatory pathways, and limiting oxidative stress [5]. Selective or focal brain cooling has been studied for aneurysm surgery, trauma, and epilepsy, demonstrating measurable reductions in intracerebral temperature without systemic complications [6].

Despite this data, neurosurgeons often employ warm irrigation based on theoretical benefits related to hemostasis or vasospasm prevention. Conversely, the rationale for cold irrigation aligns with several established neurocritical care principles: minimizing cerebral metabolic rate of oxygen (CMRO2), reducing intracranial pressure (ICP), decreasing excitotoxicity, and preventing or treating intraoperative seizures [3,4].

Given the absence of prior comprehensive narrative reviews, this article synthesizes the available data regarding the impact of irrigation temperature in cranial neurosurgery.

Methods

This narrative review utilized a literature search in PubMed, ProQuest, and ScienceDirect databases to identify relevant publications on intraoperative irrigation temperature and its neurosurgical applications. Keywords included combinations of “cold irrigation”, “warm irrigation”, “craniectomy”, “intracranial hypertension”, and related procedural terms. A total of 26 publications were reviewed by each author independently. No quantitative synthesis was performed due to heterogeneity and a lack of comparable studies. Table 1 defines temperature-related terms used in this review.

**Table 1:** Temperature ranges and common terms

Term	Definition	Source
Warm irrigation: synonymous with “hot irrigation”	>37°C	[7]
Physiologic irrigation: synonymous with “body temperature irrigation”	Approximately 37°C	[7]
Room temperature irrigation	Approximately 22-24°C	[7]
Cold irrigation	Approximately 20-22°C	[7]
Mild hypothermia	Core body temperature 32-35°C	[8]
Moderate hypothermia	Core body temperature 28-32°C	[8]
Severe hypothermia	Core body temperature less than 28°C	[8]

## Review

Overview of irrigation fluids in neurosurgery

Neurosurgeons routinely use one of three major irrigation solutions: normal saline, lactated Ringer's (LR), or artificial cerebrospinal fluid (aCSF). The choice of irrigant composition, not temperature, has historically received the most scientific attention. Isotonic normal saline (often antibiotic-impregnated) is the most cost-effective and widely available choice, making it highly used and frequently required by institutional policy for neurosurgical procedures [9]. Currently, there is limited research on the ideal temperature of irrigation in neurosurgical procedures [10].

Benefits of hypothermia on the CNS

Hypothermia attenuates multiple secondary injury pathways that contribute to neuronal death following acute neurological insult. Across experimental and clinical settings, including traumatic brain injury, ischemic stroke, cardiac arrest, and neurosurgical manipulation, cooling has consistently demonstrated neuroprotective effects through coordinated suppression of metabolic demand, excitotoxicity, inflammation, and oxidative stress [5]. These mechanisms are directly relevant to brain surgery, where exposed neural tissue is vulnerable to ischemia, retraction, and metabolic stress. A principal mechanism of hypothermic neuroprotection is the reduction of cerebral metabolic demand. Cooling lowers cerebral consumption of oxygen and glucose, preserves adenosine triphosphate (ATP) stores, and delays cellular energy failure during ischemia or trauma [4]. This metabolic suppression improves ischemic tolerance and limits secondary injury in marginally perfused tissue, a scenario frequently encountered during cranial surgery.

The clinical translation of these benefits varies by condition and cooling strategy. Importantly, focal cortical cooling via cold irrigation differs fundamentally from systemic hypothermia, providing localized neuroprotection without systemic complications [11].

Diffuse Cerebral Ischemia Following Cardiac Arrest

Anoxic brain damage is the major complication of cardiac arrest that strongly influences recovery. The only available therapy to improve neurological recovery is mild therapeutic hypothermia (32-34°C), and every effort should be made to institute therapy in patients who qualify, as cardiac arrest with widespread cerebral ischemia frequently leads to severe neurologic impairment. In a multicenter randomized controlled trial with blinded assessment of the outcome, patients who had been resuscitated after cardiac arrest due to ventricular fibrillation were randomly assigned to undergo therapeutic hypothermia (target temperature 32-34°C, measured in the bladder) over a period of 24 hours or to receive standard treatment with normothermia. The primary endpoint was a favorable neurologic outcome within six months after cardiac arrest; secondary endpoints were mortality within six months and the rate of complications within seven days. In patients who have been successfully resuscitated after cardiac arrest due to ventricular fibrillation, therapeutic mild hypothermia increased the rate of a favorable neurologic outcome and reduced mortality [12].

The "Frozen Kid" Analogy: A Lesson in Neuroprotection

The neuroprotective potential of hypothermia is vividly illustrated in the phenomenon of cold-water drowning. There are numerous documented cases of children submerged in frozen water for prolonged periods who are resuscitated with little to no neurological deficit. One report specifically details a child who survived after 147 minutes of submersion without any neurological repercussions [13]. It is postulated that this is possible due to the combined effects of the cold-induced diving reflex and rapid hypothermia. Immersion in cold water triggers bradycardia and peripheral vasoconstriction, prioritizing cerebral and myocardial perfusion, while rapid core cooling suppresses cerebral metabolism and reduces oxygen demand. These effects are amplified in children, who cool more quickly because of a higher surface-area-to-volume ratio, producing a transient hypometabolic "mini-hibernation" state that extends ischemic tolerance and preserves neurologic viability until rescue [13].

Why, then, do we not routinely apply this principle to the operating room? During the resection of a glioblastoma or a metastasis, the "penumbra" of the resection cavity (the brain tissue immediately surrounding the tumor) is subjected to micro-ischemia from retraction, cautery, and vessel sacrifice. This tissue is effectively "drowning" in a metabolic crisis. Irrigating this tissue with warm fluid is analogous to keeping a drowning victim warm; it accelerates the cellular death spiral. Conversely, cold irrigation offers a method of selective brain cooling (SBC), delivering the metabolic benefits of hypothermia directly to the vulnerable tissue without the systemic complications of whole-body cooling.

Anti-Excitotoxic and Anti-Inflammatory Impacts of Hypothermia

Beyond metabolic effects, hypothermia exerts potent anti-excitotoxic and anti-inflammatory actions. Cooling suppresses presynaptic release of excitatory neurotransmitters such as glutamate and dopamine, downregulates NMDA (N-methyl-D-aspartate) and AMPA (α-amino-3-hydroxyl-5-methyl-4-isoxazole-propionate) receptor activity, and limits intracellular calcium influx. These effects reduce neuronal hyperexcitability and downstream cell death [14]. In parallel, hypothermia lowers concentrations of pro-inflammatory cytokines, including interleukin (IL)-1β, tumor necrosis factor-alpha (TNF-α), and IL-6, inhibits matrix metalloproteinase activity, and preserves blood-brain barrier (BBB) integrity [14]. Collectively, these actions mitigate cerebral edema and reduce the risk of hemorrhagic transformation in injured tissue [3,4].

Mechanistic studies have further identified activation of the nuclear factor erythroid 2-related factor 2/antioxidant response element (Nrf2/ARE) pathway as a key mediator of hypothermic neuroprotection. Upregulation of the Nrf2/ARE pathway increases transcription of endogenous antioxidant enzymes, reducing oxidative stress and neuronal apoptosis. Hypothermia disrupts both mitochondrial and receptor-mediated apoptotic cascades, decreases activation of pro-death signaling proteins, and prolongs neuronal survival under conditions of metabolic stress [14].

Hypothermia also suppresses the formation of reactive oxygen species and enhances endogenous antioxidant defenses, including increased activity of superoxide dismutase and glutathione peroxidase, along with reduced lipid peroxidation. These molecular effects translate into lower intracranial pressure, reduced secondary brain swelling, and improved histopathological and functional outcomes in preclinical models and select patient populations [5].

Summary

Taken together, the neuroprotective effects of hypothermia are robust, multifactorial, and well supported across multiple models of brain injury. When applied focally through cold irrigation, these benefits can be leveraged at the surgical interface without exposing patients to the systemic risks of global hypothermia, such as coagulopathy, arrhythmias, and immunosuppression [6].

Harmful effects of hyperthermia on the CNS

Hyperthermia, even when mild, has well-established deleterious effects on cerebral physiology and is consistently associated with worsened neurological outcomes [2]. For each 1°C increase in temperature above 37°C, the odds of poor neurological outcome increase by 2.2-2.3 fold [15]. Elevated brain temperature amplifies secondary injury pathways, including cerebral edema, excitotoxicity, metabolic failure, and neuronal death, particularly in tissue already subjected to ischemia, trauma, or surgical manipulation.

Cerebral Edema, BBB Disruption, and Intracranial Hypertension

Hyperthermia promotes cerebral vasodilation, increasing cerebral blood volume and predisposing to cerebral edema. In parallel, elevated temperature increases BBB permeability through the upregulation of matrix metalloproteinases (MMP-2 and MMP-9), which facilitates the leakage of plasma proteins and fluid into the extracellular space, resulting in vasogenic edema [16]. These effects increase intracranial pressure, thereby elevating the risk of ischemia, infarction, and herniation in vulnerable patients.

These mechanisms are particularly relevant in neurosurgical settings, where BBB integrity may already be compromised by tumor infiltration, trauma, or surgical devascularization. Under such conditions, even transient increases in local temperature may exacerbate edema formation and secondary injury.

Local brain temperatures frequently exceed core temperatures by as much as 2°C in injured brains due to hypermetabolism in injured areas and "cerebral thermopooling"-problems in dissipating excess heat as a result of local edema formation and vascular blockage [17].

Metabolic Stress, Seizure Propensity, and Secondary Injury

Hyperthermia increases cerebral metabolic demand by accelerating biochemical reaction rates, neuronal firing, and transmembrane ion fluxes [16]. This heightened metabolic state increases oxygen and glucose consumption at a time when perfusion may be impaired, leading to metabolic mismatch and cellular stress. Focal hyperthermia further lowers seizure threshold by enhancing neuronal excitability, increasing the risk of overt or subclinical seizures, and contributing to secondary brain injury [2]. Patients presenting with seizures, or those with a seizure history prior to brain surgery, would presumably have a lower seizure threshold for intraoperative seizures, especially with intra-axial lesions requiring cortical manipulation; such patients have traditionally been treated by neurosurgeons with an extra dose of seizure medication prior to incision, and may be the biggest beneficiaries of proactive cool irrigation.

In the intraoperative setting, these effects are especially concerning, as seizures under general anesthesia may be silent, yet still capable of exacerbating neuronal injury and postoperative deficits.

Cellular Injury, Protein Denaturation, and Excitotoxicity

At the cellular level, hyperthermia induces protein denaturation and disrupts cell membrane integrity, both of which contribute directly to neuronal injury. Loss of protein tertiary structure triggers cellular stress responses and can overwhelm protective mechanisms such as heat shock proteins. Concurrently, disruption of neuronal membranes increases ionic leakage, impairs membrane potential stability, and compromises cellular homeostasis [2].

Hyperthermia also exacerbates excitotoxic injury by increasing glutamate release while simultaneously reducing astrocytic glutamate uptake. Excess synaptic glutamate leads to overactivation of NMDA and AMPA receptors, resulting in massive calcium influx and activation of apoptotic and necrotic cell death pathways [2].

Summary

Hyperthermia worsens cerebral edema, increases metabolic stress, lowers seizure threshold, and directly accelerates neuronal injury through protein denaturation, membrane disruption, and excitotoxicity. These effects directly oppose the goals of neuroprotection and tissue preservation during brain surgery. Given the well-established harms of hyperthermia on the central nervous system, practices that increase local cortical temperature, such as warm irrigation, are physiologically difficult to justify.

Arguments supporting warm irrigation

The rationale for warm irrigation in brain surgery is mainly based on two proposed benefits: improved hemostasis and prevention of vasospasm. These arguments are largely generalized from non-neurosurgical contexts and do not withstand physiologic or evidentiary scrutiny when applied to the central nervous system.

Hemostasis

Warm irrigation is frequently justified by its perceived coagulative benefit and its role in preventing systemic hypothermia. In general surgery trauma patients, even mild hypothermia (core body temperature between 32-34°C) can impair platelet function and slow enzymatic reactions within the coagulation cascade, thereby worsening bleeding [1]. However, this reasoning conflates the effects of systemic hypothermia in abdominal surgery with localized cortical temperature modulation in brain surgery. Additionally, it is important to note that no randomized controlled trials have shown that mild systemic hypothermia increases intracranial bleeding risk [4].

The brain differs fundamentally from other organs in its metabolic profile and tolerance to temperature variation. Localized cooling of exposed neural tissue reduces metabolic demand and does not impair systemic coagulation pathways, which are governed primarily by hepatic enzyme kinetics and circulating platelet function at core body temperature. Importantly, studies examining selective brain cooling during craniotomy demonstrate meaningful reductions in intracerebral temperature without altering core body temperature. Additionally, brain temperature returned to baseline after cessation of cold irrigation in 5.3 ±1.5 minutes, demonstrating the transient and reversible nature of focal cooling [6]. Intuitively, it makes sense to use focal hypothermia for focal brain surgery, as the part of the brain being operated upon is the area most at risk of seizure, edema, and BBB disruption.

Additionally, neurosurgical hemostasis is driven predominantly by mechanical techniques and microvascular control, and there is no evidence demonstrating improved hemostasis with warm irrigation in cranial surgery.

Vasospasm Prevention

Another commonly cited argument for warm irrigation is the prevention of vasospasm [18]. Because cold irrigation can induce vasoconstriction, some theorize it may provoke or worsen vasospasm. However, this concern is speculative and unsupported by clinical evidence. There are no studies demonstrating that cold irrigation induces clinically significant intraoperative vasospasm in brain surgery. Many neurosurgeons have historically used deliberate mild intraoperative hypothermia. This appears counterintuitive for concerns about intraoperative vasospasm from focal hypothermia [19]. Accounts of observers covering themselves with blankets to stay warm in the operating room are described anecdotally by Dr. Javed Siddiqi (author), who trained under Dr. Mahmut Gazi Yasargil in 1996-1997 at the University of Arkansas as his skull base and vascular fellow.

Evidence from aneurysm surgery, where vasospasm occurrence and consequences are greatest among all types of neurosurgical procedures, does not support this theoretical risk. The large multicenter Intraoperative Hypothermia for Aneurysm Surgery Trial (IHAST) randomized 1001 “good-grade patients” (World Federation of Neurological Surgeons score, I-III) undergoing aneurysm clipping to intraoperative systemic hypothermia (target 33°C) versus normothermia (target 36.5°C) and found no difference in the incidence of ischemic events (clinical vasospasm) or overall outcomes between groups [20].

A systematic review of intraoperative hypothermia for aneurysm surgery similarly found no increased risk of vasospasm with cooling, with 34 of 577 (6%) participants with hypothermia and 32 of 581 (5.5%) participants without hypothermia experiencing clinical vasospasm (temporary deficits) [21]. Further, multiple other systematic reviews provide additional support that intraoperative mild hypothermia does not increase vasospasm risk [17,22]. Interestingly, studies have shown that the presence of increased core body temperature is actually associated with vasospasm occurrence [23], which further debunks the argument that warm irrigation is better for vasospasm prevention. 

Finally, when intraoperative vasospasm does occur, it is readily visible to the surgeon and can be reliably treated with topical vasodilators such as papaverine [24]. In contrast, increasing cortical temperature to avoid a theoretical risk of vasospasm introduces well-documented harms (including increased metabolic demand, lowered seizure threshold, and exacerbation of secondary brain injury). From a risk-benefit perspective, warm irrigation offers no demonstrable protective advantage.

Chronic Subdural Hematoma Recurrence

The only cranial neurosurgical scenario with high-level evidence favoring warmer over cooler irrigation is in the reduction of chronic subdural hematoma (cSDH) recurrence after burr-hole evacuation. A multicenter randomized controlled trial demonstrated that body-temperature irrigation (37°C) reduced six-month recurrence requiring reoperation to 6%, compared with 14% using room-temperature irrigation (22°C) in a cohort of 541 adults. While the relative risk reduction appears impressive (64%), the absolute risk reduction is only 8% (14% vs 6%), meaning approximately 13 patients would need to be treated with warmed irrigation to prevent one recurrence. The authors' proposed mechanism is physiologically plausible: warmer irrigation increases solubility and clearance of hematoma degradation products while avoiding local hypothermia-induced coagulopathy, thereby limiting reaccumulation [25].

However, this finding must be interpreted within its clinical context. Risk of cSDH recurrence is driven predominantly by membrane biology rather than irrigation temperature alone, particularly the presence of organized inner and outer membranes that perpetuate inflammatory exudation. The variability between different cSDHs and the fact that an increasing number of these are also now being treated with middle meningeal artery embolization as a standard adjunct imply that the temperature of the irrigation is not the whole pathophysiological story.

Summary

The use of warm irrigation is a residue from liver surgery, where focal cool irrigation may limit the coagulation factors secreted by the liver, resulting in increased bleeding. However, this phenomenon is not extrapolatable to the brain, where thromboplastin is released from primary or iatrogenic brain damage, so mitigating that injury with hypothermic irrigation would make intuitive sense, and could be reinforced by coagulation profile measurement during brain surgery.

Arguments supporting cold irrigation

Hypothermia is neuroprotective because it decreases CMRO2, limits oxidative stress, reduces apoptosis, and improves functional outcomes in multiple injury models [14]. Targeted hypothermia is used in neurocritical care practice to control intracranial pressure (ICP) when all other measures have failed [3,4]. Cooling produces vasoconstriction and reduces cerebral blood volume, transiently lowering ICP, which is beneficial when the brain is being manipulated during surgery.

Targeted Neuroprotection without Systemic Risk

Selective brain cooling with cold irrigation allows localized modulation of cortical temperature at the surgical interface while maintaining normothermia at the systemic level and in the rest of the non-manipulated brain. This is simple to implement intraoperatively and provides targeted neuroprotection (reduces cerebral metabolic demand, suppresses excitotoxic cascades, and stabilizes cellular membranes) to the area being manipulated by the surgeon, without the risks associated with systemic hypothermia [26].

Suppression and Prevention of Intraoperative Seizures

Cold irrigation is an established first-line abortive therapy for cortical seizures during open cranial procedures [3]. There is significant evidence from clinical studies and reviews showing that focal cortical cooling during epilepsy surgery effectively and rapidly aborts intraoperative seizures, reducing the need for anticonvulsant drugs. The first management of intraoperative seizures in epilepsy patients is cold irrigation to the operative site, which is known to abort seizures faster than any pharmacologic management [27]. Clinical case series have demonstrated that chilled saline irrigation effectively suppresses epileptiform activity during epilepsy surgery. Ablah et al. reported that application of chilled (4°C) saline directly to the cortex during cortical mapping and surgical resection significantly reduced interictal spike frequency, providing direct human evidence that focal cooling decreases cortical excitability; they found no significant reduction in the epileptic spike frequency with room temperature normal saline irrigation [28].

These findings are particularly relevant in the intraoperative setting, where seizures under general anesthesia may be present but clinically silent. We suspect that a good portion of patients slow to wake up from anesthesia after cranial surgery are actually postictal from undiagnosed silent seizures. Cold irrigation offers an immediate, reliable, and low-risk method of preventing and suppressing seizure activity during cortical manipulation.

Timing-Dependent Neuroprotection and Injury Mitigation

Experimental data further support the importance of early and localized cooling. Yokobori et al. demonstrated that mild hypothermia initiated before craniotomy in a rat model of acute subdural hematoma significantly reduced biomarkers of neuronal and glial injury (ubiquitin carboxyl-terminal hydrolase L1 (UCH-L1) and glial fibrillary acidic protein (GFAP)), decreased histopathologic cell death, and minimized injury volume compared with normothermia or delayed cooling [29].

These findings underscore that early hypothermia before secondary injury cascades are fully engaged confers the greatest neuroprotective benefit. Cold irrigation allows neurosurgeons to apply this principle intraoperatively, delivering early focal cooling during periods of high metabolic stress.

Summary

Cold irrigation provides targeted, immediate, and physiologically sound neuroprotection during brain surgery. It suppresses cortical excitability, reduces metabolic stress, mitigates secondary injury, and does so without exposing patients to the risks of systemic hypothermia. In contrast to warm irrigation, which offers no proven benefit, cold irrigation aligns with established neuroprotective principles and has direct clinical utility. Its routine use during cranial surgery is both rational and evidence-supported.

## Conclusions

Warm irrigation is commonly used by neurosurgeons, although evidence supporting its use is limited and largely theoretical. In contrast, basic neuroscience and clinical data demonstrate neuroprotective advantages of cold irrigation: reduction of cortical temperature, suppression of neuronal hyperexcitability, metabolic downregulation, reduction of oxidative injury, and improved tolerance of marginally perfused tissue. Evidence supporting warm irrigation for vasospasm prevention or chronic subdural hematoma recurrence is weak, inconsistent, and is not a good analogy for intraparenchymal brain manipulation as necessary in brain tumor surgery. When vasospasm does occur, it is visible and can easily be treated with topical papaverine. Intraoperative seizures are more common than intraoperative vasospasm and can be silent when they occur. Therefore, prevention of seizures should take precedence over theoretical prevention of vasospasms.

Selective brain cooling with cold irrigation represents an immediately available, low-risk, and physiologically sound strategy that aligns with established neuroprotective principles used in neurocritical care and experimental neurobiology. High-quality randomized trials are still needed to determine the gold standard for temperature irrigation. However, the cumulative evidence strongly suggests cold irrigation as the more rational and protective default in neurosurgical practice. Routine adoption of cold irrigation has the potential to reduce secondary brain injury, minimize silent intraoperative seizures, and improve outcomes across a wide spectrum of cranial procedures. Finally, the claim that warm irrigation improves hemostasis in brain surgery is not backed by neuroscience or supported by data. This practice persists from inherited surgical dogma and reflects habit and tradition, not evidence, as the brain is distinct from all other organs in the body and should not be treated as the liver.
